# Distinct patterns of pigment development underlie convergent hyperpigmentation between nocturnal and diurnal geckos (Squamata: Gekkota)

**DOI:** 10.1186/s12862-020-01604-9

**Published:** 2020-03-27

**Authors:** Aaron H. Griffing, Tony Gamble, Aaron M. Bauer

**Affiliations:** 1 Department of Biological Sciences, Marquette University, P.O. Box 1881, Milwaukee, WI 53201 USA; 2 Milwaukee Public Museum, 800 W. Wells Street, Milwaukee, WI 53233 USA; 3 Bell Museum of Natural History, University of Minnesota, Saint Paul, MN 55108 USA; 4 Department of Biology and Center for Biodiversity and Ecosystem Stewardship, Villanova University, 800 Lancaster Avenue, Villanova, PA 19085 USA

**Keywords:** Crypsis, Embryology, Gekkonidae, Melanophore, Squamate, Temporal niche

## Abstract

**Background:**

Evolutionary transitions in temporal niche necessitates specialized morphology, physiology, and behaviors. Diurnal, heliothermic squamates (lizards and snakes) that bask require protection from ultraviolet radiation (UV) that can damage internal organs such as the brain, viscera, and gonads. Many smaller squamates have accomplished this protection by hyperpigmentation of the peritoneum and subcutaneous dorsum. Typically, nocturnal species do not require these protections from ultraviolet light. However, some nocturnal species that exhibit extreme crypsis may be exposed to sunlight and UV and require some means of mediating that damage. One such species is *Gekko* (*Ptychozoon*) *kuhli*, a nocturnal, arboreal gecko that uses extreme crypsis to blend in with tree bark. Hiding motionless on tree trunks leaves geckos exposed to sunlight during the day. Thus, we predict that *G. kuhli* will have independently evolved a hyperpigmented phenotype. To investigate this hypothesized association between temporal niche, behavior, and morphology, we characterized adult subcutaneous pigment for eight gecko species and embryonic pigment accumulation for a subset of four of these species, exhibiting diverse temporal niche and thermoregulatory behaviors. We predicted that nocturnal/potentially-heliothermic *G. kuhli* would exhibit hyperpigmentation of internal structures like that of diurnal/heliothermic geckos. We further predicted that embryonic pigment accumulation of *G. kuhli* would resemble that of diurnal/heliothermic as opposed to nocturnal/thigmothermic geckos.

**Results:**

We found that temporal niche and thermoregulatory behavior predicted the degree of subcutaneous pigment in the eight gecko species examined. We demonstrate that *G. kuhli* accumulates pigment extremely early in embryonic development, unlike a diurnal/heliothermic gecko species, despite having a similar adult phenotype.

**Conclusions:**

The evolution of hyperpigmentation in *G. kuhli* is likely an adaptation to limit damage from occasional daytime UV exposure caused by crypsis-associated basking behavior. *Gekko kuhli* achieves its hyperpigmented phenotype through a derived developmental pattern, not seen in any other lizard species investigated to date, suggesting novel temporal differences in the migration and/or differentiation of reptilian neural crest derivatives.

## Background

Temporal niche, also known as diel activity niche, is an important aspect of the biology of an organism, necessitating the evolution of specialized morphology, physiology, ecology, and behavior (e.g. [19, 41, 56, 72]). For example, many diurnal ectotherms thermoregulate through basking behavior (i.e. heliothermy), whereas nocturnal ectotherms thermoregulate through contact with surfaces of different temperatures (i.e. thigmothermy; [1, 16, 51]). Temporal niche appears to be phylogenetically conserved across major tetrapod clades [2] and thus many adaptations to specific temporal niches (diurnal, nocturnal, crepuscular, or cathemeral) are shared among closely related species. Despite its conservation in tetrapod evolutionary history [2], several squamate clades do exhibit temporal niche turnover. The crown group of geckos (Infraorder Gekkota) are hypothesized to be ancestrally nocturnal, with reversals to diurnality occurring in at least 10 lineages [2, 24, 76]. Many of these lineages exhibit an array of diurnal-specialized adaptations, most notably eye morphologies, with oil droplets which aid in light filtering and spectral tuning [9, 55, 70, 76], concaviclivate temporal fovea to aid in binocular vision [57, 71], and ovoid retinal pigmented epithelia (RPE) to aid in light filtering and absorption [31, 65].

Another phenotype that is typically correlated with diurnal temporal niche in vertebrates is the hyperpigmentation of internal structures, such as the overlaying connective tissues of the brain, gonads, subcutaneous dorsum, and peritoneum [13, 34, 39, 50]. These dense collections of melanophores are hypothesized to protect internal structures from injurious and mutagenic UV radiation, which heliotherms encounter more frequently than thigmotherms [12, 13, 38, 50, 52]. Though heliothermy is correlated with hyperpigmentation of internal structures, some gecko species exhibit a disconnect between thermoregulatory behavior and temporal niche. For example, *Sphaerodactylus* geckos (Sphaerodactylidae) are primarily diurnal, but are active underneath leaf-litter and are thus thigmothermic [33]. Alternatively, *Strophurus* geckos (Diplodactylidae) are primarily nocturnal, yet occasionally bask during daylight hours [25]. This “occasionally-heliothermic” classification is supported by *Strophurus* exhibiting hyperpigmented peritonea [25].

Parachute geckos (Subgenus *Ptychozoon*) of the genus *Gekko* comprise 12 described species which inhabit dipterocarp forests of southeast Asia [11, 32, 68, 81]. This clade is characterized, in part, by a suite of specialized traits, including expanded trunk folds, expanded caudolateral folds, and elaborate interdigital webbing, which allow for a gliding predator escape behavior [8]. Following Russell’s [59] step-wise hypothesis, gliding behavior through these elaborate cutaneous folds was exapted from use of the folds to reduce shadows (i.e. cryptic behavior) and thus, in conjunction with cryptic coloration, conceal the animal from predators [5, 28–30, 49, 58, 64, 67, 73]. Though chiefly nocturnal, *Gekko* (*Ptychozoon*) *kuhli* can occasionally be found on exposed tree trunks and branches during the day [28–30, 66]. This is likely a byproduct of cryptic behavior, as remaining motionless on tree trunks and branches throughout the day may result in exposure to direct sunlight and suggests an occasionally-heliothermic thermoregulatory classification. As mentioned previously, prolonged exposure to direct sunlight necessitates adaptations to tolerate higher temperatures and increased UV. We therefore hypothesize that behavioral crypsis, as implemented by *G. kuhli*, can lead to occasional heliothermy and the correlated phenotypic changes despite exhibiting a nocturnal temporal niche. To further investigate this hypothesized association between temporal niche, behavior, and pigment phenotype, we qualitatively characterized subcutaneous (fascial, visceral, and peritoneal) pigment for eight gecko species exhibiting diverse temporal niche and thermoregulatory behaviors. We predicted that nocturnal/potentially-heliothermic *G. kuhli* would exhibit hyperpigmentation of internal structures like that of diurnal/heliothermic geckos. Furthermore, to characterize patterns of pigment accumulation through embryonic development, we examined embryos at various stages of development from four gecko species exhibiting all combinations of temporal niche and thermoregulatory character states. We predicted that embryonic pigment accumulation of *G. kuhli* should also resemble that of diurnal/heliothermic as opposed to nocturnal/thigmothermic geckos.

## Results

**Fig. 1:**
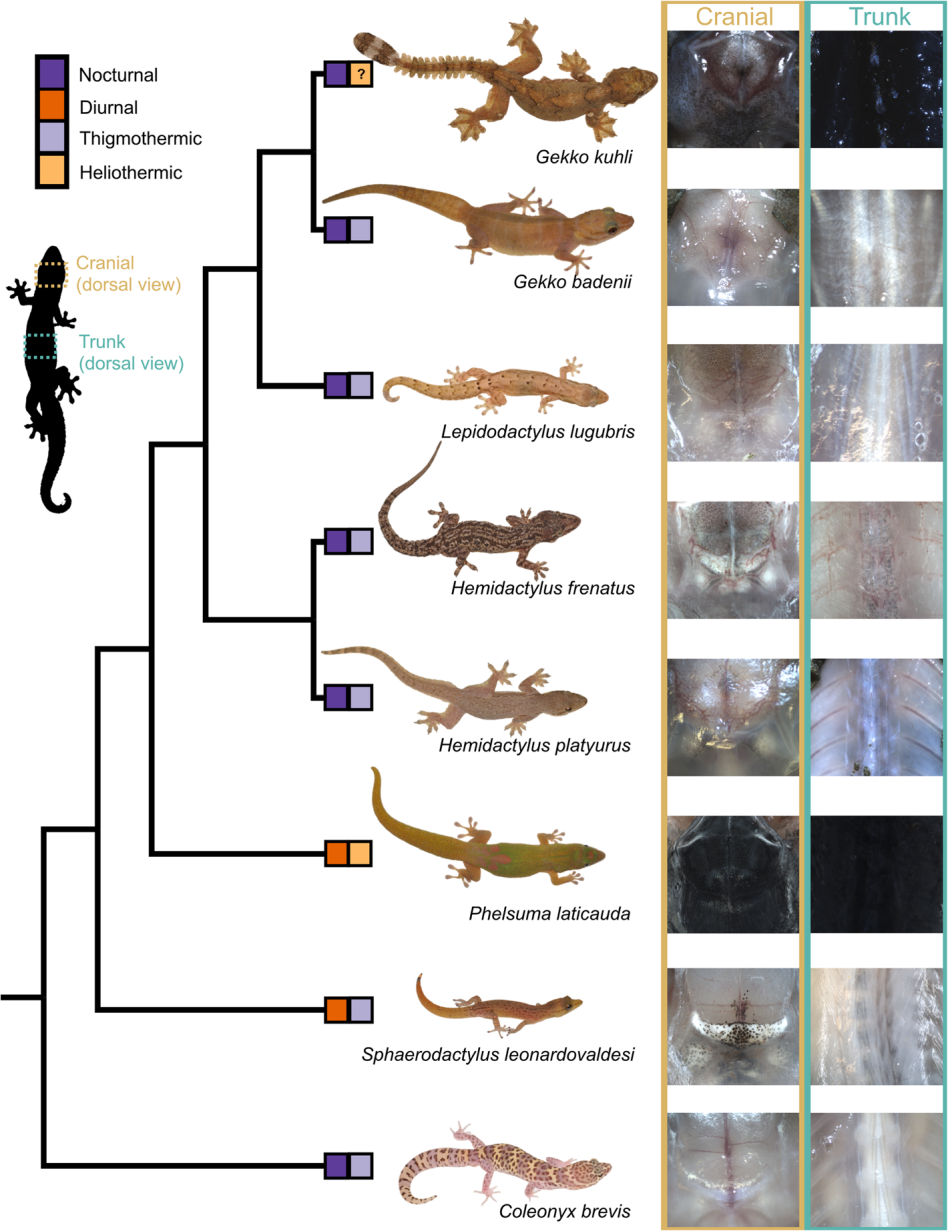
Convergent evolution of subcutaneous dorsal hyperpigmentation in the geckos. Phylogenetic relationships of eight gekkotan taxa, exhibiting a variety of temporal niche and basking behavior character states, following the topology of Gamble et al. [24]. Dorsal views of the skinned parietal region (brown) and the mid trunk region (green) correspond to adjacent tips of the phylogeny. Gecko photographs: Stuart Nielsen

**Fig. 2:**
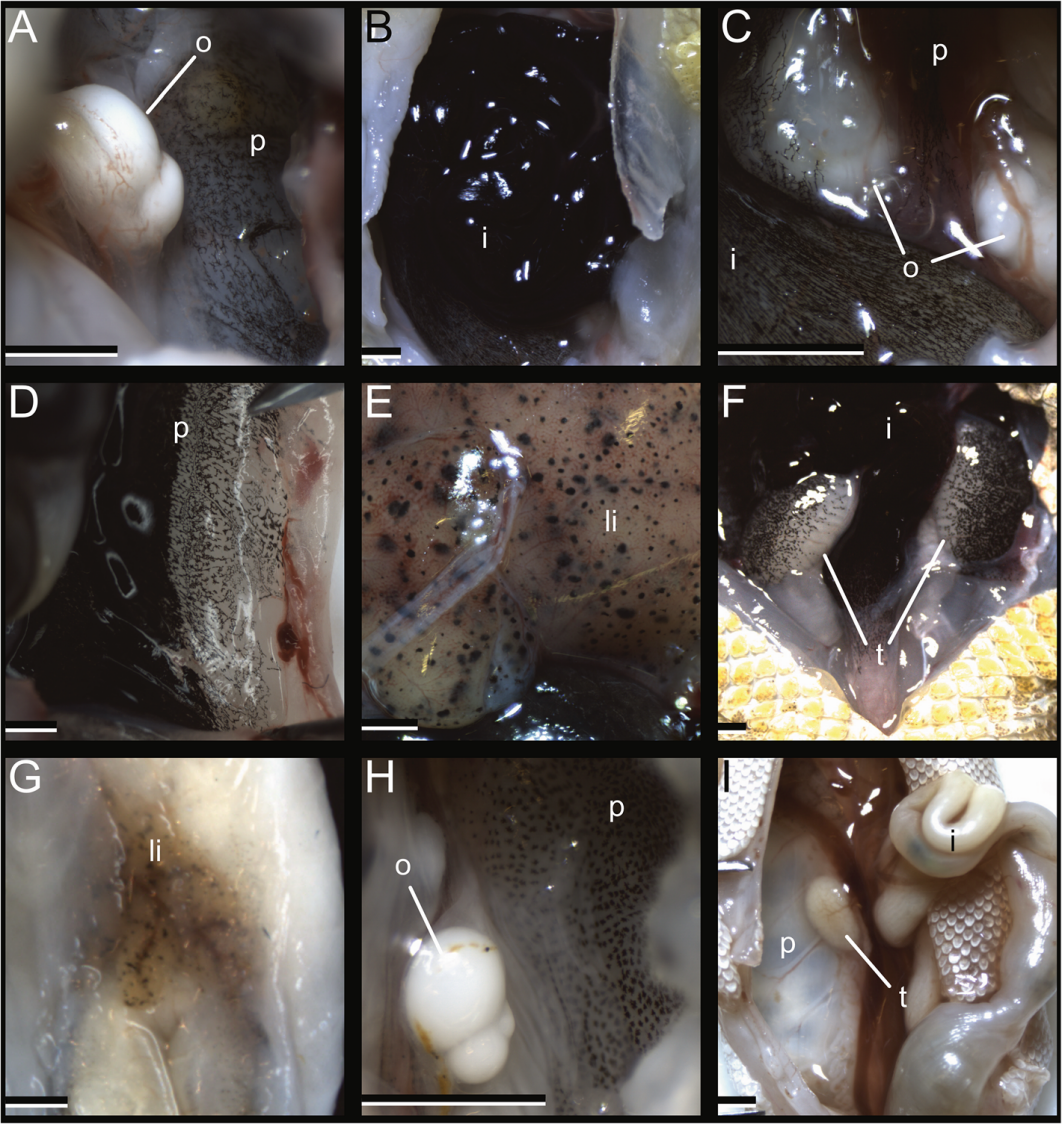
Diversity of pigmented visceral serosae and peritonea in geckos. **a** ovaries and lightly pigmented peritoneum of *G. kuhli*. **b** Black intestines of *H. platyurus*. **c** Lightly pigmented ovaries and peritoneum of *H. platyurus*. **d** Black and lightly pigmented peritoneum of *P. laticauda*. **e** Lightly pigmented liver of *P. laticauda*. **f** Black intestines and darkly pigmented testes of *P. laticauda*. **g** Lightly pigmented liver of *S*. *leonardovaldesi*. **h** Ovaries and lightly pigmented peritoneum of *S*. *leonardovaldesi*. **i** Completely unpigmented viscera and peritoneum of *C. brevis* which is identical to all other species investigated lacking internal melanophores. i, intestines; li, liver; o, ovaries; p, peritoneum; t, testes. Scale bars =1 mm

Adult nocturnal/thigmothermic species exhibited no pigment on the subcutaneous dorsal fascial surface (Fig. 1). Of these five species, only *Hemidactylus platyurus* exhibits pigment on the inside of the body cavity — the gonadal serosa is lightly pigmented, the peritoneum is lightly pigmented, and the intestinal serosa is black (Fig. 2; Table 1). The only diurnal/thigmothermic species, *Sphaerodactylus leonardovaldesi*, exhibits no pigment on the subcutaneous dorsal fascial surface, with the exception of a lightly pigmented area posterior to the parietals (Fig. 1). Internally, *S*. *leonardovaldesi* exhibits a lightly pigmented peritoneum and liver (Fig. 2; Table 1). The diurnal/heliothermic *Phelsuma laticauda* exhibits a black subcutaneous dorsal fascia surface along the skull, through the parietal region and along the trunk, shifting from black to dark pigmentation near the pelvic region (Fig. 1). Internally, *P. laticauda* exhibits a lightly pigmented liver, darkly pigmented gonadal serosa, both light and black areas of the peritoneum, and black intestinal serosa (Fig. 2; Table 1). Finally, the nocturnal/potentially-heliothermic *Gekko kuhli* exhibits a black subcutaneous dorsal fascial surface along the trunk and a darkly pigmented parietal region and remaining skull (Fig. 1). Internally, *G. kuhli* exhibits a lightly pigmented peritoneum and no pigment on the remaining viscera Fig. 2; (Table 1).

**Table 1:** Hyperpigmentation in geckos

Species	Temporal niche / thermoregulatory behavior	Fascial Pigment (Anterior, Posterior)	Peritoneal Pigment	Visceral Pigment
*G. kuhli*	N/H	2, 3	1	liver (0), stomach/ intestines (0), gonads (0)
*G. badenii*	N/T	0, 0	0	liver (0), stomach/ intestines (0), gonads (0)
*L. lugubris*	N/T	0, 0	0	liver (0), stomach/ intestines (0), gonads (0)
*H. frenatus*	N/T	0, 0	0	liver (0), stomach/ intestines (0), gonads (0)
*H. platyurus*	N/T	0, 0	1	liver (0), stomach/ intestines (3), gonads (1)
*P. laticauda*	D/H	3, 3	1–3	liver (1), stomach/ intestines (3), gonads (2)
*S. leonardovaldesi*	D/T	1, 0	1	liver (1), stomach/ intestines (0), gonads (0)
*C. brevis*	N/T	0, 0	0	liver (0), stomach/ intestines (0), gonads (0)

Pigment levels are coded as follows: 0, no melanophores or no pigment; 1, scattered melanophores or lightly pigmented; 2, many melanophores or darkly pigmented; and 3, opaque coating of melanophores or black. D, diurnal; H, heliothermic; N, nocturnal; T, thigmothermic. Names of organs are listed with their associated serosal pigment level

**Fig. 3:**
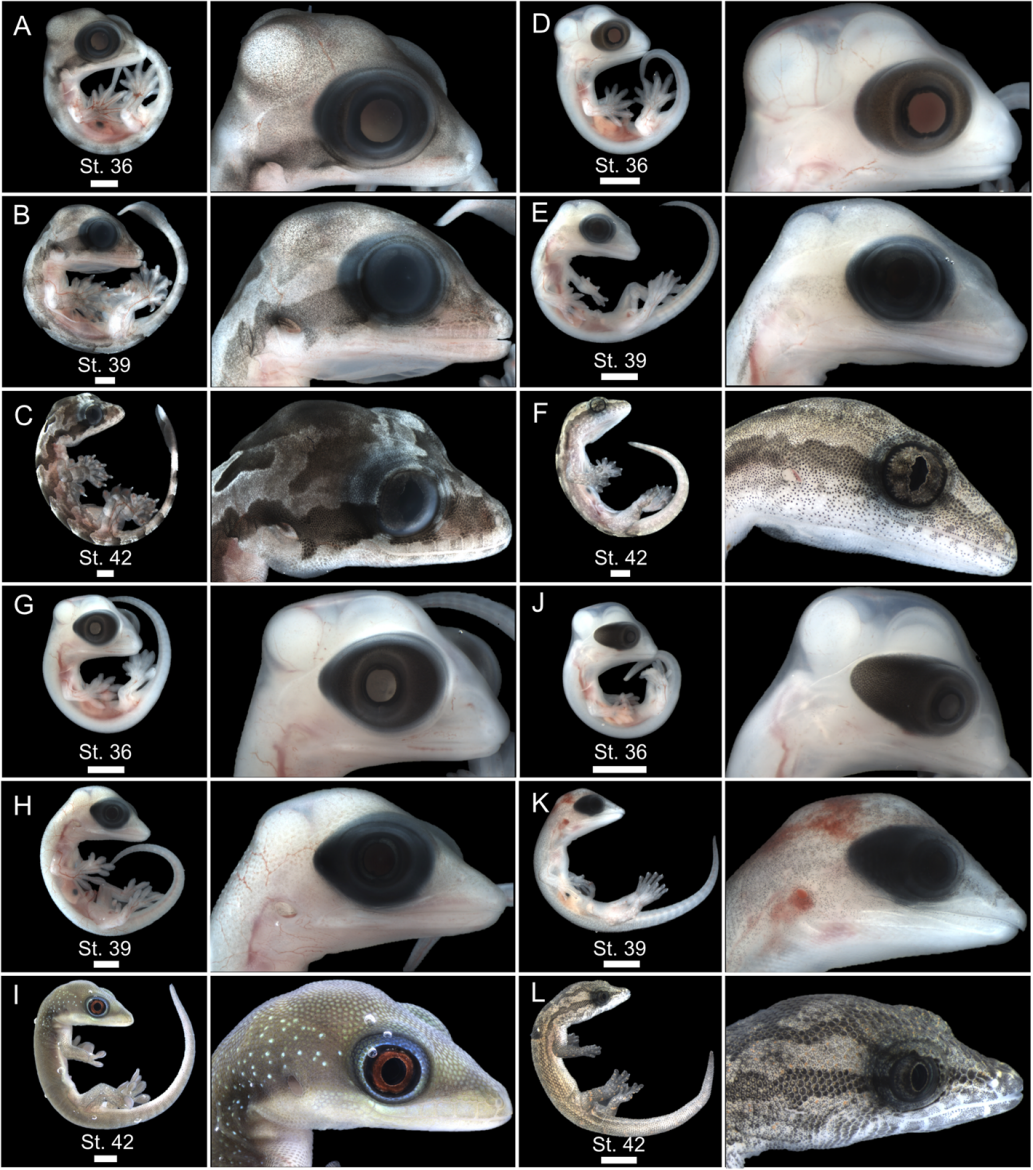
Embryonic comparison between two nocturnal gekkonids: potentially heliothermic, cryptic *Gekko kuhli* and thigmothermic, non-cryptic *Lepidodactylus lugubris*. Note the early accumulation of dorsal and craniofacial pigment in *G. kuhli* while none is visible in *L. lugubris*. Row **a** Lateral view of whole *G. kuhli* embryos, stages 29–36. Row **b** Lateral view of *G. kuhli* embryos craniofacial region, stages 29–33. Row **c** Lateral view of *G. kuhli* embryos craniofacial region, stages 34–36. Row **d** Lateral view of whole *L. lugubris* embryos, stages 29–36. Row **e** Lateral view of *L. lugubris* embryos craniofacial region, stages 29–33. Row **f** Lateral view of *L. lugubris* embryos craniofacial region, stages 34–36. White arrows indicate area of pigment accumulation. Scale bars = 2 mm

**Fig. 4:**
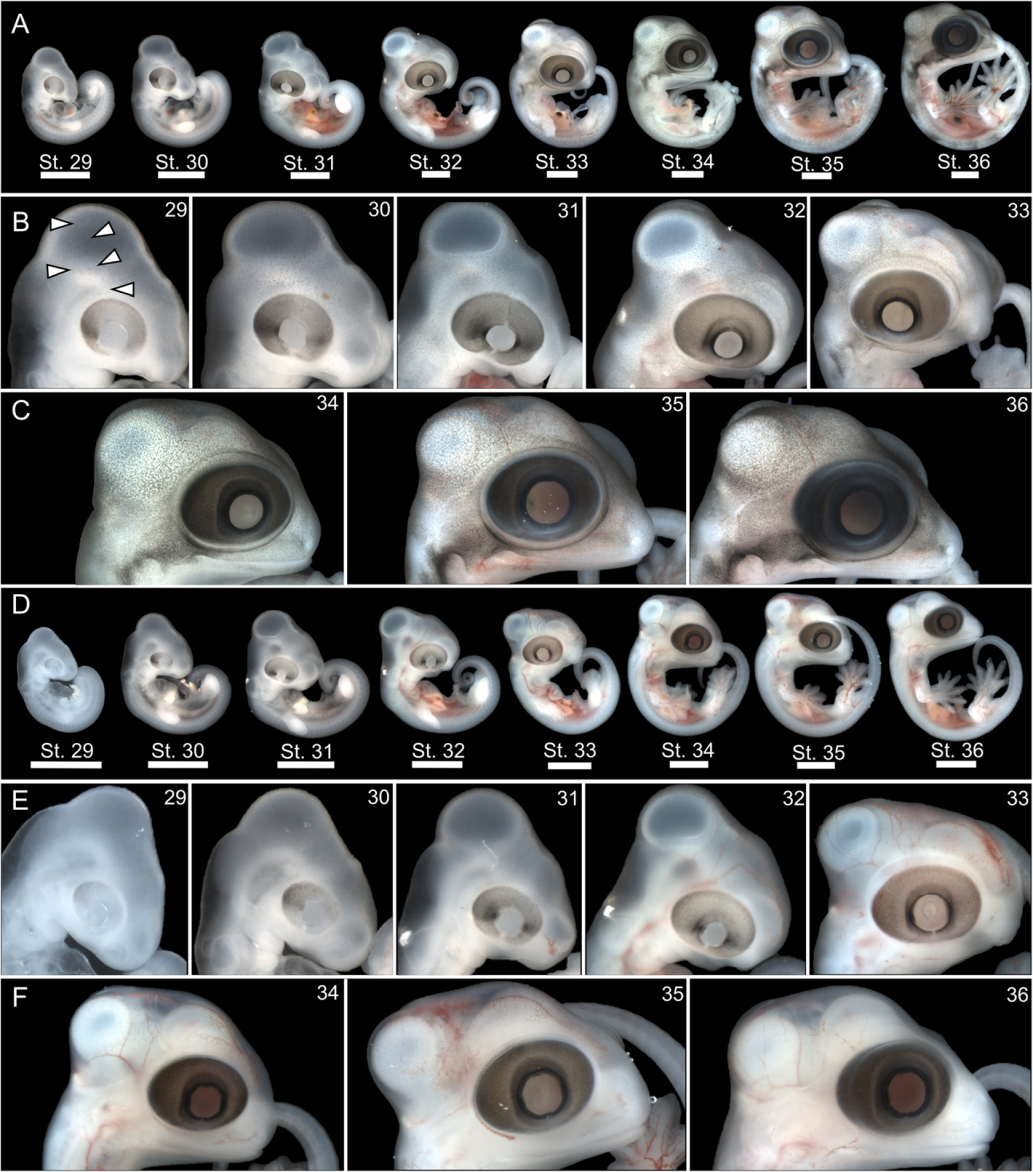
Three embryonic stages of four gecko species showcasing lack of dorsal and craniofacial pigment (stage 36), early visible accumulation of dorsal and craniofacial pigment (stage 39), and near-hatching dorsal and craniofacial pigment (stage 42). *Gekko kuhli* stages 36 (**a**), 39 (**b**), and 42 (**c**). *Lepidodactylus lugubris* stages 36 (**d**), 39 (**e**), and 42 (**f**). *Phelsuma laticauda* stages 36 (**g**), 39 (**h**), and 42 (**i**). *Sphaerodactylus macrolepis* stages 36 (**j**), 39 (**k**), and 42 (**l**). Scale bars = 2 mm

The first external pigment cells to accumulate in all gecko embryos are restricted to the RPE (Fig. 3 [31];). Accumulation of melanophores, outside of the RPE, during embryonic development of *G. kuhli* begins shortly after oviposition Stage 29 (i.e. mid-limb bud stage; Fig. 3a,b). These initial sparse accumulations are located in the epidermis along the dorsum, outside of the developing optic tectum, and adjacent to the eye (Fig. 3a,b). At Stage 30, sparse melanophore accumulation spreads over the pharyngeal arches and the majority of the craniofacial region (Fig. 3a,b). By Stage 31, sparse accumulation has reached the forelimbs and the pigment accumulation along the dorsum and craniofacial region is more dense (Fig. 3a,b). From Stage 31 to Stage 36, melanophore accumulation becomes denser and covers the entire surface of the embryo and begins to resemble the color pattern of near-hatchling *G. kuhli*: little pigment on the ventral surface, dense pigment on the dorsum creating faint chevron patterns, and dense pigment adjacent to the eye forming a dorsolateral stripe (Fig. 3a–c). By comparison, embryos of *L. lugubris* do not exhibit visible melanophores outside of the RPE during equivalent stages of development (Stages 29–36; Fig. 3d–f). Indeed, regardless of temporal niche or basking behavior, all gecko embryos examined, with the exception of *G. kuhli*, lacked visible accumulation of pigment outside of the RPE until Stage 38–39 (Fig. 4). Sparse pigment accumulates along the center of the dorsum in Stage 38, and eventually spreads to the craniofacial region in Stage 39 (Fig. 4). During Stage 39, the pigment faintly resembles the eventual pattern of the near-hatchling animal and is colocalized with the epidermal papillae that will give rise to scales (i.e. Stage 42; Fig. 4).

## Discussion

As predicted, *G. kuhli* exhibits darkly pigmented to black subcutaneous dorsal fascia while none of the other nocturnal gecko species examined exhibit dorsal fascia pigmentation (Fig. 1). As expected, and similar to *G. kuhli*, the diurnal/heliothermic gecko, *P. laticauda*, also exhibits black dorsal fascia pigmentation (Fig. 1). Furthermore, the diurnal/thigmothermic gecko, *S*. *leonardovaldesi* exhibits an intermediate phenotype: light dorsal fascia pigmentation near the braincase (Fig. 1). The only previous in-depth investigations into gecko subcutaneous pigmentation was performed by Duncker [20–22], who examined 20 species. Duncker, who noted the extreme pigmentation of *Phelsuma* spp., also described fascial pigmentation in the largely nocturnal but often heliothermic *Tarentola* spp. [61] as well as pigmented nervous and vascular tissue of the largely nocturnal but heliothermic *Ptyodactylus hasselquistii* [3, 79]. The peritonea and the serosa of various visceral elements are pigmented in *G. kuhli*, *P. laticauda*, and *S*. *leonardovaldesi*. Duncker [21] reported pigmented intestine of *G. kuhli*, though we did not find this. There are multiple explanations for this discrepancy. First, Duncker’s *G. kuhli* specimens may represent a different species from the *G. kuhli* specimens we examined, and *G. kuhli*, like many other species in the genus, may be a species complex comprised of multiple undescribed taxa [11, 17]. Second, *G. kuhli* is a widespread species in Southeast Asia [11] and there may be intraspecific, regional variation. Interestingly, the gonads, intestines, and peritoneum of *H. platyurus* are pigmented. *Hemidactylus platyurus*, similar to *G. kuhli*, is known to parachute, use elaborate body folds to aid in cryptic behavior, and is occasionally known to bask [35, 59, 62, 64, 69], supporting the hypothesis that nocturnal geckos with cryptic diurnal behavior are exposed to ultraviolet radiation more frequently than other nocturnal gecko species and therefore require specialized protection. Indeed, the nocturnal and behaviorally cryptic, *Uroplatus fimbriatus* exhibits pigmentation in the digestive tract and the cloaca [77]. These hyperpigmented patterns represent similar evolutionary routes to protect the various internal delicate organs from UV and suggests species can take similar evolutionary paths to achieve similar functional goals in different structures [7, 44, 75]. When compared to *G. kuhli*, the lower degree of subcutaneous pigmentation exhibited by *H. platyurus* may be explained by behavioral differences between the species. Though *H. platyurus* is indeed behaviorally cryptic, anecdotal evidence suggests its behavioral crypsis is less effective than that of *G. kuhli* [64]. Taylor [66] noted that *Gekko* (*Ptychozoon*) *lionotus* can be reluctant to move from their cryptic positions and will flee only following “considerable disturbance,” whereas *H. platyurus* flee from similar positions with little disturbance [62]. Field observations also suggest that *H. platyurus* regularly use crevices in trees, rocks, gardens, and houses near human activity as day-time hiding locations ([10, 64, 66]; pers. observation in Philippines by AHG and TG) and are less likely to be exposed during the day compared to *G. kuhli*. This preliminary association between cryptic behavior and hyperpigmented phenotype, though promising, requires further corroboration through robust taxon sampling.

Vertebrate pigment cells are ultimately derived from neural crest cells, which begin migrating from the neural tube during the 6–9 somite stage in *Chamaeleo calyptratus* [18, 40]. In avian reptiles and mammals, these unpigmented precursor cells migrate to the epidermis where mature melanocytes synthesize pigment which can then be deposited to epidermal appendages such as hair or feathers [63, 82]. Alternatively, non-avian reptiles, amphibians, and fishes produce three common types of chromatophores (xanthophores, iridophores, or melanophores) as well as more phylogenetically restricted pigment cell types (e.g. cyanophores, leucophores), for which developmental trajectories are still not well understood [4, 23, 36, 48]. Despite this diversity, there is considerable conservation in molecular pathways responsible for melanocyte and chromatophore development [15, 47, 48]. The overall spatial pattern of pigment accumulation exhibited by *G. kuhli* appears similar the other gecko species examined — pigment accumulates along the epidermis overlaying the developing brain and the dorsum, adjacent to the anterior portion of the neural tube. However, the early onset temporal pattern of pigment development exhibited by *G. kuhli* has not been described in any other gecko species to date [26, 31, 37, 43, 45, 74, 78, 80, 83], let alone other lizard species (e.g. [18, 42, 46, 53]). Heterochrony, specifically an early onset of melanophore migration, maturation, or pigment production, may explain the hyperpigmented adult phenotype of *G. kuhli* (Fig. 3). However, the same cannot be said for the hyperpigmented adult phenotype of *P. laticauda* or the intermediate pigmented phenotype of *S. macrolepis* (Fig. 4) highlighting how distinct developmental programs can lead to convergent phenotypes [60, 75]. Further studies of squamate neural crest development are necessary to investigate interspecific variation in melanophore migration, specifically with regards to hyperpigmented peritonea or dorsal fascia [18, 54].

## Conclusions

Herein we propose the hypothesis that behavioral crypsis can lead to situations which require heliothermic adaptation. *Gekko kuhli*, a nocturnal gliding gecko with behavioral crypsis, exhibits a degree of subcutaneous pigmentation that is typically only seen in diurnal/heliothermic geckos such as *Phelsuma* spp. Another behaviorally cryptic, nocturnal gecko, *H. platyurus*, exhibits similar elaborate pigmentation on some viscera but not the dorsal fascia. Further investigations into this connection between thermoregulatory behavior and pigment phenotypes should test whether *G. kuhli* and *H. platyurus* can tolerate higher temperatures and are exposed to less ultraviolet damage than sister taxa with less pigment. Furthermore, *G. kuhli* appears to exhibit hyperpigmentation throughout most of postovipositional embryonic development, a developmental pattern which differs from other geckos, including heliothermic species. Due to this unique pattern, we suggest *G. kuhli* as a model to study temporal changes to typical reptilian patterns of neural crest derivative migration.

## Methods

We qualitatively characterized subcutaneous (fascial, visceral, and peritoneal) pigment for six gekkonid gecko species exhibiting a diversity of temporal niche and thermoregulatory behaviors: *Gekko kuhli* (nocturnal/potentially-heliothermic), *Gekko badenii* (nocturnal/thigmothermic), *Lepidodactylus lugubris* (nocturnal/thigmothermic), *Hemidactylus frenatus* (nocturnal/thigmothermic), *Hemidactylus platyurus* (nocturnal/thigmothermic), and *Phelsuma laticauda* (diurnal/heliothermic). This taxon sampling allows us to compare dorsal fascial pigmentation of 3 of the 4 possible character state combinations and spanning the diversity of the Gekkonidae [24]. We also compare two outgroups: one sphaerodactylid (*Sphaerodactylus leonardovaldesi*) and one eublepharid (*Coleonyx brevis*) which exhibit diurnal/thigmothermic and nocturnal/thigmothermic charter states, respectively. Each individual (*N* = 8) was euthanized humanely using MS222 following Conroy et al. [14], skinned and eviscerated to reveal subcutaneous pigment, and finally observed and photographed using a Nikon SMZ 74ST stereoscope. We characterized degree of pigmentation following Bauer [6]: no melanophores (no pigment), scattered melanophores present (lightly pigmented), many melanophores present (darkly pigmented), and complete opaque coating of melanophores present (black).

We collected eggs from captive colonies of four gecko species exhibiting all combinations of character states to observe embryonic patterns of pigment development: 49 embryos of *G. kuhli* (nocturnal/potentially-heliothermic), 141 embryos of *L. lugubris* (nocturnal/thigmothermic), 13 embryos of *P. laticauda* (diurnal/heliothermic), and 26 embryos of *Sphaerodactylus macrolepis* (diurnal/thigmothermic). Because embryos of *S*. *leonardoveldesi* were unavailable, we collected embryos of *S. macrolepis* as a congeneric proxy. We collected embryos (*N* = 229) following protocols described by Griffing et al. [27]. To briefly summarize, we removed embryos from eggs using #5 watchmaker’s forceps while immersed in diethyl pyrocarbonate (DEPC) treated, RNase free 1% phosphate-buffered saline, and visualized and photographed using a Nikon SMZ 74ST stereoscope. As geckos exhibit interspecific variation between the precise time points (days post-oviposition; DPO) of developmental stages (Noro et al., 2009 [26, 37, 74, 80, 83];), we discretized and assigned developmental stages based on external morphology using previous embryonic staging series of geckos rather than characterizing by DPO [26, 80].

## Data Availability

The data (stereoscope images) supporting the results of this article are available in the FigShare repository, DOI: 10.6084/m9.figshare.11914965, 10.6084/m9.figshare.11915001, 10.6084/m9.figshare.11915007, and 10.6084/m9.figshare.11915013.All specimens used in this study are housed at the Department of Biological Sciences, Marquette University (Milwaukee, Wisconsin).
